# A Comparison Between Dexmedetomidine and Clonidine as Adjuvants to Levobupivacaine in Labour Analgesia

**DOI:** 10.7759/cureus.20237

**Published:** 2021-12-07

**Authors:** Shruti Kabi, Reetu Verma, Dinesh Singh, Premraj Singh, Jyotsna Agarwal, Brij Bihari Kushwaha, Ajay K Chaudhary, Nisha Singh

**Affiliations:** 1 Department of Anaesthesiology and Critical Care, King George's Medical University, Lucknow, IND; 2 Department of Anaesthesiology and Critical Care, King George’s Medical University, Lucknow, IND; 3 Department of Obstetrics and Gynaecology, King George's Medical University, Lucknow, IND

**Keywords:** labour analgesia, dexmedetomidine, clonidine, levobupivacaine, neuraxial analgesia, epidural analgesia

## Abstract

Background

The epidural analgesia technique is effective for labor analgesia and combinations of various local anesthetics with lipophilic opioids like fentanyl are used. However, fentanyl can cause an increased incidence of pruritus, urinary retention, nausea, vomiting, giddiness, shivering, and respiratory depression. Dexmedetomidine and clonidine are selective alpha 2 agonists with analgesic properties and have been used via the neuraxial route with local anesthetics for the same without the side effects of fentanyl. Thus, the primary objective was to assess and compare the analgesic efficacy of the two-drug combinations by the visual analog scale (VAS) score.

Methods

Fifty-four primigravida women were randomly allocated in two groups of 27 each and were given an initial bolus of 10 mL of 0.125% levobupivacaine with dexmedetomidine 0.5 ug/kg in Group A and with clonidine 1 μg/kg in Group B. Subsequently, each patient received a background infusion rate of 10 mL/h, a bolus dose of 5 ml, and a lock-out interval of 10 min via a patient-controlled-analgesia (PCA) pump. The blood pressure, heart rate, and severity of pain using VAS were assessed. Durations of the stages of labor, rate of instrumental delivery, and cesarean section, side effects, maternal sedation, and neonatal Apgar scores were also recorded.

Results

VAS scores in both the groups progressively decreased to <3 by 15 min with significant differences at five, 10, 15, and 120 min being lower in group A. Onset of analgesia and time for maximum analgesia was significantly shorter in group A. There was a significant decrease in hemodynamic parameters from baseline in both groups. The fall in heart rate was significantly greater in Group A and at almost all the time intervals after baseline, diastolic blood pressure (DBP) was also lower in group A. Maternal motor blockade scores, the intensity of maternal sedation, the incidence of maternal complications, the duration of the first and second stage of labor, the rate of instrumental delivery and cesarean section, total analgesic dose and PCA bolus requirement, and neonatal Apgar scores did not show a significant difference between the two groups.

Conclusion

Both dexmedetomidine and clonidine provide hemodynamically stable labor with a fall in heart rate and maternal blood pressure in the initial hours. Dexmedetomidine has the advantage of faster onset of analgesia and time for maximum analgesia.

## Introduction

Epidural analgesia is one of the most common methods of pain relief during labor in clinical practice. Dilute solutions of local anesthetics given by the epidural route provide adequate analgesia and do not block the motor nerves of the uterus, ensuring that the parturient has normal uterine contraction and bearing down ability [1]. Thus it allows labor to proceed unhindered while the parturient walks normally.

The mainstay of the block is to achieve maximum analgesia while avoiding or minimizing the motor blockade effect of the local anesthetic. Adjuvants such as fentanyl prolong the duration of a motor-sensory block, hasten the onset of action, and decrease the side effect or toxicity of local anesthetics by decreasing the total cumulative dose required. However, fentanyl can cause an increased incidence of pruritus, urinary retention, nausea, vomiting, giddiness, shivering, and respiratory depression [2].

Both clonidine and dexmedetomidine are alpha-2 adrenergic agonists that have analgesic as well as sedative properties when used as an adjuvant in regional anesthesia and hence are being tried as newer adjuvants for labor analgesia [3]. Both have been shown to reduce the requirements of local anesthetics and enhance the analgesic effects without increasing the incidence of side effects [4-5].

However, to the best of our knowledge, no previous study has compared clonidine with dexmedetomidine for labor analgesia. Hence, we planned a double-blind, randomized, comparative study with an aim to compare the effects of both these drugs when used epidurally as an adjuvant to levobupivacaine in labor analgesia. The primary objective was to assess and compare the analgesic efficacy of the two-drug combinations by visual analog scale (VAS) score. The secondary objectives were to assess and compare the two-drug combinations for onset time of analgesia, quality of analgesia, motor block in lower extremities by modified Bromage score, hemodynamic effects/stability, maternal side effects/complications, the total volume of anesthetic solution used, and bolus frequency, duration of each labor stage, cesarean delivery rate, and neonatal outcome.

## Materials and methods

After ethical approval (approval no.1364/ethics/19) was given by the institutional medical ethics committee, this randomized, double-blind, comparative study was conducted in the department of obstetrics and gynecology, King George’s Medical University, Lucknow, over one year (September 2019-2020).

Fifty-four primigravida, otherwise hemodynamically stable patients between 18 and 35 years of age with body mass index (BMI) less than 35 kg/m^2^, of American Society of Anaesthesiologists' (ASA) grade II [6], with healthy term pregnancy in stage I of labor with vertex presentation were included. Any patients with preeclampsia, eclampsia, pregnancy-induced hypertension, systemic hypertension, diabetes mellitus, heart disease, previous lower segment cesarian section (LSCS) or any absolute indication for LSCS, allergy to study drugs, infection at the site of epidural catheterization, coagulopathy, evidence of spinal cord injury, patients not giving consent, and those with extremes of body weight or height were excluded from the study.

After taking informed written consent, all 54 patients were randomly divided into two groups of 27 using the list of computer-generated numbers sealed in envelopes. The procedure was started in the first stage of labor when regular uterine contraction appeared and cervical dilatation was about 3-4 cm. After complete preoperative evaluation, ECG, and pulse oximetry, a blood pressure cuff was attached. Intravenous access was achieved with a 20G peripheral cannula and each patient was pre-loaded with 10 ml/kg bodyweight Ringer’s lactate solution or Sterofundin 10-15 minutes before induction of epidural analgesia over a period of 30 min. The procedure was performed in the sitting position. With proper aseptic and antiseptic precautions, under local anesthesia (2% lignocaine), a multi-hole epidural catheter was inserted at L3-4 intervertebral space with an 18G Touhy needle using the loss of resistance technique to air. The catheter was placed 3-4 cm in the epidural space in the cephalic direction. A test dose of 3 ml 2% lignocaine with 1:200000 epinephrine was administered to exclude intravenous or subarachnoid catheter placement after negative aspiration for CSF and blood along with the absence of tachycardia. If no toxicity reaction appeared, epidural analgesia was started.

The solutions were prepared by a separate anesthetist, and the anesthetist who administered the drug and the recorded response was blinded to group allocation.

Group A received an initial bolus of 10 ml of 0.125% levobupivacaine with dexmedetomidine 0.5 ug/kg. Group B received an initial bolus of 10 ml of 0.125% levobupivacaine with clonidine 1 μg/kg. Subsequently, in each patient, the mixed solution was infused continuously by a patient-controlled-analgesia pump [Perfusor® fm (MFC), B Braun, Malaysia] at a background infusion rate of 10 ml/h, rescue bolus dose of 5 ml, and lock-out interval of 10 min.

Monitoring

After giving the drugs, the level of analgesia was checked by a pinprick with a 23G needle in the mid-clavicular line every five min till the level of T10 was achieved. The severity of pain was assessed before the block and at 15, 30, 45, and 60 mins and then at 30 min intervals using VAS (0 = no pain, 10 = worst possible pain experienced). Motor block was assessed bilaterally after attainment of maximum sensory block and then at hourly intervals using the modified Bromage scale (0 = no block, 1 = inability to raise extended the leg, 2 = inability to flex the knee, and 3 = inability to flex the ankle and foot). Maternal blood pressure, heart rate, and oxygen saturation were measured non-invasively every five minutes for 15 minutes, then every 15 minutes for 45 minutes, then every 30 minutes for 180 minutes, or the delivery of the fetus, whichever was early. Bradycardia (HR less than 50 beats/minute) was treated by 0.005 mg/kg atropine i.v injection, which may be repeated as needed. Any hypotension (systolic BP <90 mmHg or mean arterial pressure <65 mmHg) was treated by intravenous infusion of normal saline and intravenous injection of mephentermine 6 mg, which was repeated if needed.

We defined the onset of analgesia as the time from epidural drug injection to the time of recording a VAS ≤ 3 during active uterine contraction. and time to reach maximum analgesia as time from epidural drug injection to time of recording the lowest VAS of either 0, 1, or 2 during active uterine contraction.

Side effects such as nausea, vomiting, headache, hypotension, pruritus, hypersensitivity reaction, respiratory depression, drowsiness, fever, and shivering were noted. The level of sedation was evaluated using the Ramsay sedation score (RSS) [7] (1 = patient anxious, agitated, or restless, 2 = patient cooperative, oriented, tranquil, and alert, 3 = patient responds to commands, 4 = asleep but with brisk response to a light glabellar tap or loud auditory stimulus, 5 = asleep, sluggish response to a light glabellar tap or loud auditory stimulus, 6 = asleep, no response). Excessive sedation was defined as RSS value >4. Values were recorded every 60 minutes during labor.

Post-partum patients were asked to rate their overall quality of analgesia for labor and delivery from 0 to 2 (where 0 = worse than expected, 1 = as expected, and 2 = better than expected). The quality of natural expulsive efforts at the time of delivery was asked to the obstetrician (0 = poor, 1 = fair, 2 = moderate, 3 = good). The total dose of local anesthetic required, duration of the first and second stages of labor, mode of delivery in the form of normal vaginal delivery, instrumental delivery, or cesarean section was observed. Neonatal Apgar scores at one min and five mins and side effects on the neonate were noted.

Statistical analysis

Data were analyzed using software version SPSS 12.0 (SPSS Inc., Chicago, IL). Demographic data were analyzed using analysis of variance. The unpaired t-test and chi-square test were used wherever appropriate. A sample size of 54 was determined using a previous study by Kaur S et al. [8] with the power of study of 90% and a confidence interval of 95%. Quantitative data were expressed as mean ± SD, and discrete (categorical) data were summarized as proportions and percentages (%). Standard tests of significance were applied to determine the P-value. P<0.05 was considered significant.

## Results

The demographic profiles of the patients in both groups were comparable with regards to age, weight, and body mass index (P>0.05) (Table 1). At baseline, all the hemodynamic parameters and VAS scores of the two groups were matched.

**Table 1:** Distribution of Subjects According to Anthropometry and Pregnancy Characteristics GA = Gestational Age

Variable	Group A		Group B		t-value	p-value
	Mean	SD	Mean	SD		
Age (years)	25.00	3.54	25.63	3.52	0.66	0.515
Height (cm)	157.18	3.05	158.67	3.26	1.73	0.089
Weight (Kg)	56.96	3.24	55.00	4.26	1.90	0.063
GA in days	263.19	8.28	266.44	9.64	-1.33	0.188

Sensory level up to T-10 was achieved in all patients.

Primary outcome

**Figure 1:**
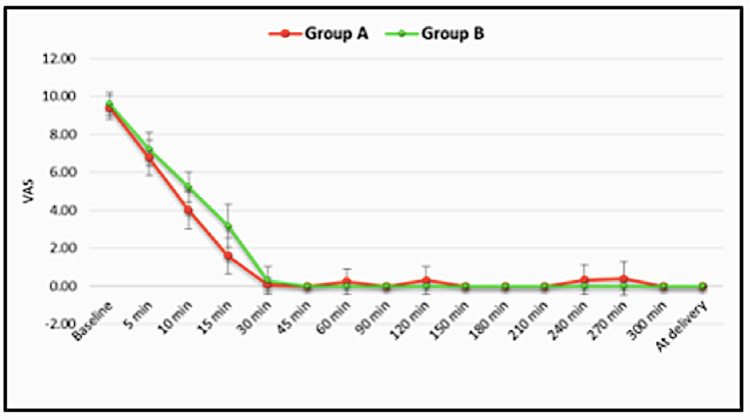
Intergroup Comparison of VAS at Various Time Points VAS = Visual Analog Scale

Administration of the bolus dose resulted in a progressive decrease in VAS that decreased to a mean of 1.59±9.5 in group A and 3.19±1.14 in group B at 15 minutes. From 30 minutes till the end of labor, the VAS was comparable in the two groups. The significant differences in mean VAS were observed among the groups at 5 min (p=0.044), 10 min (p<0.001), 15 min (p<0.001), and 120 min (p=0.039) (Figure 1).

Comparison between the studied groups showed a statistically significant difference in time of onset of analgesia and time for maximum analgesia with both being significantly faster in group A (p<0.001 for both) (Table 2).

**Table 2:** Intergroup Comparison of Onset and Maximum Analgesia Time Max = Maximum

Onset & Max Analgesia time (min)	Group A		Group B		t-value	p-value
	Mean	SD	Mean	SD		
Onset	11.185	1.301	15.629	2.096	9.36	<0.001
Max analgesia	14.630	1.244	18.180	2.180	7.35	<0.001

Secondary outcome

The mean Bromage score was almost zero in both the groups at all the time points except for one incidence of motor involvement (Bromage score= 1) in group B without any significant difference.

**Figure 2:**
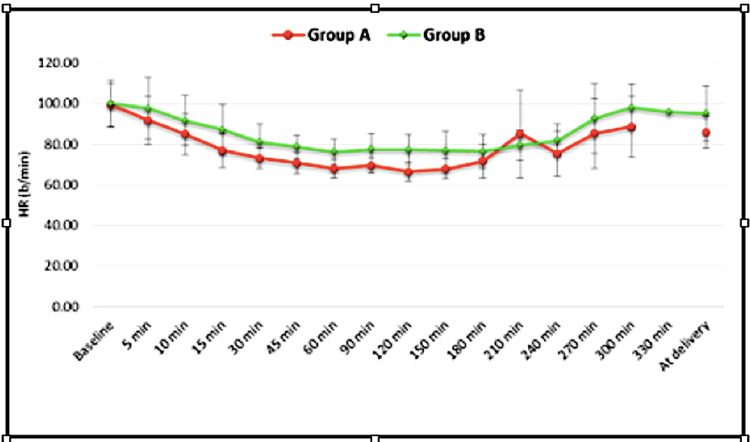
Intergroup Comparison of Heart Rate at Various Time Points HR = Heart Rate

After commencement of epidural drug administration, there was a gradually progressively decreasing trend in mean heart rate at all intervals up to 180 min in both the groups. Thereafter, the heart rate remained almost stable. However, the fall in heart rate was significantly greater in group A with significant differences between 15 minutes and 180 minutes (p<0.05) and then at delivery (p=0.007) (Figure 2).

**Figure 3:**
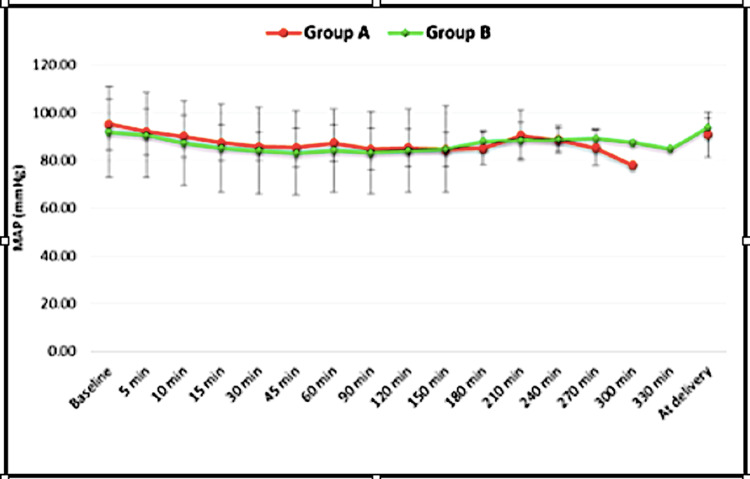
Intergroup comparison of Mean Arterial Pressure at Various Time Points MAP = Mean Arterial Pressure

Systolic blood pressure (SBP) reduced in both the groups with a gradually descending trend till nearly 180 min. Thereafter, it was a relatively stable trend till delivery. No significant differences in SBP were observed. In almost all the time intervals after baseline, diastolic blood pressure (DBP) was lower in Group A, with significant differences in DBP occurring at 45 min (p<0.001), 120 min (p=0.001), 150 min (p<0.001), 180 min (p=0.018), and at the time of delivery (p=0.018). No significant intergroup difference in MAP was observed up to 300 min (p=0.003), which was clinically insignificant (Figure 3).

Statistical analysis revealed that the incidence of side effects was comparable in both groups (Table 3).

**Table 3:** Intergroup Comparison of Side Effects

Side effects	Group A		Group B		chi sq	p-value
	Number	Percentage(%)	Number	Percentage(%)		
Nil	23	85.2	25	92.6	Ref.	
Bradycardia	1	3.7	0	0.0	1.06	0.302
Hypotension	1	3.7	0	0.0	1.06	0.302
Motor involvement	1	3.7	0	0.0	1.06	0.302
Nausea	1	3.7	2	7.4	0.24	0.623
Non-progression	0	0.0	1	3.7	0.90	0.342

There was no significant difference in the duration of the first and second stages of labor, which remained normal in both groups. The total amount of local anesthetic agents used and the number of boluses taken were also comparable between the two groups. There were no significant fetal side effects in either group (Table 4).

**Table 4:** Intergroup Comparisons of Durations of Stages of Labour, Doses Required, and Fetal Effects

	Group A (Mean±SD)	Group B(Mean±SD)	t-value	p-value
Duration of first stage (min)	234.79±36.31	240.00±29.12	0.58	0.563
Duration of the second stage (min)	36.75±5.60	39.61±5.13	1.96	0.056
Total Dose (ml)	53.80±11.45	55.55±9.32	0.62	0.540
Number of boluses	1.17±0.71	1.33±0.73	0.82	0.418
Birth weight (kg)	2.66±0.38	2.60±0.37	0.61	0.545
APGAR at 1 min	6.75±1.03	7.13±0.69	1.49	0.144
APGAR at 5 min	8.92±0.88	9.04±0.71	0.53	0.596

The incidence of instrumental delivery and caesarian section was similar in the two groups (Table 5).

**Table 5:** Distribution of Mode of Delivery Between the Two Groups NVD = Normal Vaginal Delivery; LSCS = Lower Segment Cesarian Section

Mode of delivery	Group A (N=27)		Group B (N=27)	
	Number	percentage(%)	Number	percentage(%)
NVD	20	74.1	18	66.7
Forceps	1	3.7	3	11.1
Ventouse	3	11.1	2	7.4
LSCS	3	11.1	4	14.8

All women were satisfied with the level of analgesia they experienced. Analgesia was better than expectation (score of 2) for 96% of women in group A and 100% of women in group B (p=0.313).

The two groups did not show any statistically significant difference in the nature of expulsive efforts (p=0.613). On comparing the power of explosive efforts, we found that 66.7% of patients in group A and 69.6% in group B had good expulsive efforts (score of 3) while 29.2% in group A and 30.4% in group B had moderate expulsive efforts (score of 2).

## Discussion

Epidural bupivacaine had been used extensively in the past for providing pain relief in patients undergoing labor and delivery. However, in recent years, levobupivacaine has increasingly replaced bupivacaine because of its similar analgesic properties and decreased propensity of cardiotoxicity [9]. The concentration of levobupivacaine used in our study was 0.125% because studies show it to be nearly equipotent to bupivacaine (ratio of 0.98) [10-11].

In this study, we aimed to compare the effects of dexmedetomidine and clonidine when added to levobupivacaine for labor epidural analgesia. We used clonidine in the dose of 1 mcg/kg in loading, bolus, and infusion, which, as per the built and weight of women included in our study, approximates 50-70 mcg of clonidine. This is consistent with previous studies that have shown the effective range to be 30-100 mcg [12-14]. A study comparing four different doses of epidural dexmedetomidine for epidural labor analgesia postulated that the optimum concentration of dexmedetomidine is 0.5 μg/ml [4]. Hence, for our study, we used dexmedetomidine at the dose of 0.5 μg/kg. As per the weight and built of the patients in our study, this dose allowed the concentration to remain in the optimum and safe range.

The primary objective of our study was to compare the analgesic efficacy of dexmedetomidine and clonidine on the basis of the VAS score. We observed that in both groups, effective analgesia was achieved with VAS decreasing to less than 3 within 15 minutes of the loading dose. Significant differences were seen at 5, 10, and 120 minutes. At these intervals, the mean VAS was lower in the dexmedetomidine group. The statistical significance at 120 minutes was not clinically relevant, as the mean VAS of both groups at 120 min was less than 0.5, which is a clinically acceptable grade of analgesia.

Comparison of our present data with other literature is rather difficult, as most of the published studies have studied the two adjuvants separately using different drug administration protocols or concentrations. However, similar trends were seen in studies based on dexmedetomidine as well as clonidine [15-18].

The mean onset time of analgesia was significantly faster in the dexmedetomidine group than in the clonidine group. The time for maximum analgesia was also significantly lower in the dexmedetomidine group. The degree of reduction in the onset time of analgesia depends on the definition of onset time used in each study. We defined the onset of analgesia as the time from epidural drug injection to the time of recording a VAS ≤ 3 during active uterine contraction. Using the same definition, Kaur S et al. observed a faster onset of analgesia with 0.5 μg/kg dexmedetomidine when added to 0.125% levobupivacaine as compared to 0.125% ropivacaine in labor [8]. Their mean onset time was 11.16±1.86 minutes, which complied with our study. Ahirwar A et al. defined the onset of analgesia as the time from the first bolus dose to the time of achieving VAS <4 and found the onset in the clonidine group to be 9.89±3.50 min [19].

With regards to motor involvement, none of the patients in the clonidine group showed a motor block. In the dexmedetomidine group, only one case had motor involvement with a modified Bromage score of 1 and was statistically insignificant. Similar results were obtained by other studies [8,19]. Soliman R et al. documented a higher incidence of motor block in the dexmedetomidine group compared to fentanyl, which could be due to the higher concentration of 1 mcg/ml used by them [20].

As regards hemodynamic parameters, our results revealed a significant decrease in heart rate compared to baseline values in both groups but the decrease was significantly more in the dexmedetomidine group. Only one patient in the dexmedetomidine group had transient bradycardia that did not require any type of intervention. The SBP, DBP, and MAP in both the groups had a gradually decreasing trend till around three hours after which they remained more or less stable. DBP was comparatively lower in group A. We preloaded the patients to avoid any hypotension due to sympathectomy, and it seems that the preloading is adequate to prevent an episode of hypotension associated with the initiation of analgesia. Transient hypotension occurred in one patient receiving dexmedetomidine that responded to 250 ml fluid bolus and did not require i.v mephentermine.

The maternal expulsive efforts were comparable in both groups with most of the patients having good to moderate expulsive efforts. None of the patients in our study had poor expulsive efforts that may lead to increased incidence of assisted/cesarean deliveries. The rate of spontaneous vaginal deliveries, forceps, ventouse, and cesarean deliveries in the two groups were comparable with no statistically significant difference. The indications for cesarian section were thick meconium-stained liquor (n=3, one in group A and two in group B), non-progression of labor (n=1, in group B), non-reactive non-stress test (NRNST) (n=2, one in each group), and cord prolapse (n=1, in group A). These were in concordance with other studies [11,19-20]. However, Roelants et al. compared two different concentrations of clonidine, 1.5 ug/ml and 3 ug/ml, with sufentanyl in labor and found a higher rate of instrumental delivery in both the clonidine groups compared to the sufentanyl group [21]. A possible cause could be the higher concentration of clonidine used by them in both groups compared to our concentration.

Both dexmedetomidine and clonidine did not affect the duration of the first and second stages of labor, which were comparable in both groups. There was no statistically significant difference in the total dose of the anesthetic drug used in both groups, with boluses ranging between 0 and 3. All women in the surgery were satisfied with the quality of analgesia achieved.

We did not find any statistically significant side effects in either group. In the dexmedetomidine group, side effects were hypotension, bradycardia, motor involvement, and nausea seen in one case each while with clonidine, they were nausea, seen in two cases, and non-progression of labor, seen in one case. None of the patients in either group had any profound sedation, which also correlates with other studies [22-23]. In concordance with other studies, there was no significant difference in Apgar scores between the two groups [20-21]. Only two neonates in the dexmedetomidine group had a one-minute Apgar score of less than 6. One newborn had an Apgar of 4 due to low birth weight of 1.8 kg and a delayed, weak cry. He was intubated and shifted to the neonatal intensive care unit (NICU). The second baby had a one-minute Apgar of 5. She had a delayed cry with a five-minute Apgar of 8.

There are a number of limitations to our study. First, ours was a single-center clinical trial with a small number of participants. It is possible that differences in the reported outcomes still exist but could have been influenced by the small sample size. Second, we included only the Apgar score to assess the neonatal effects and did not use other parameters such as fetal heart tracing (FHR) and neonatal pH. Apgar scores alone are insufficient measures of neonatal depression.

## Conclusions

Both dexmedetomidine and clonidine given epidurally as an adjuvant to levobupivacaine are effective in relieving the pain and discomfort of labor. Dexmedetomidine, however, has the advantage of faster onset of analgesia and less time taken to achieve maximum analgesia. When combined with levobupivacaine as adjuvants for labor analgesia, they do not affect maternal expulsive efforts or cause any motor block that can affect ambulation. Both provide hemodynamically stable labor with a fall in heart rate and maternal blood pressure in the initial hours that remain within the normal range.
